# Restoration based on cost–benefit optimization: A grasslands pilot study

**DOI:** 10.1002/eap.70174

**Published:** 2026-01-29

**Authors:** Sarah R. Weiskopf, Toni Lyn Morelli, Tina G. Mozelewski, Alexey N. Shiklomanov, Susannah B. Lerman

**Affiliations:** ^1^ U.S. Geological Survey National Climate Adaptation Science Center Reston Virginia USA; ^2^ Department of Environmental Conservation University of Massachusetts Amherst Massachusetts USA; ^3^ U.S. Geological Survey Northeast Climate Adaptation Science Center Amherst Massachusetts USA; ^4^ Spatial Informatics Group‐Natural Assets Laboratory Pleasanton California USA; ^5^ NASA Goddard Space Flight Center Greenbelt Maryland USA; ^6^ USDA Forest Service Northern Research Station Amherst Massachusetts USA

**Keywords:** biodiversity modeling, conservation, global change, grasslands, restoration

## Abstract

Ecological restoration is essential to meeting global biodiversity conservation goals. Given limited conservation budgets, deciding where to restore habitat is a key challenge for the coming decade. We developed a spatially explicit framework to optimize ecological restoration site selection by integrating land use history, species distributions, and economic costs. The framework includes the following steps: identify potential restoration area based on relevant environmental measures like land use; identify species of interest; calculate restoration benefits by modeling habitat and climate suitability and estimating reduced extinction risk associated with restoring a particular land parcel based on a modified species–area relationship; aggregate benefits across species; and compare to parcel‐level land acquisition costs. We applied linear programming to maximize conservation benefit/restoration cost ratios to identify optimized restoration sites. We illustrate this approach using a case study for highly threatened grassland ecosystems in the Great Plains region of Kansas, USA. We selected five grassland animal species (greater prairie chickens [*Tympanuchus cupido*], lesser prairie chickens [*Tympanuchus pallidicinctus*], swift fox [*Vulpes velox*], pronghorn [*Antilocapra americana*], and regal fritillary [*Speyeria idalia*]) as indicators of restoration benefit across taxa. For the indicator species that we chose, shortgrass and mixed‐grass prairies had the highest conservation benefit to cost ratio. Setting a minimum restoration threshold for each habitat type allowed us to identify high‐priority tallgrass prairie sites. Despite increasing interest in ecological restoration, optimizing restoration site selection is challenging because one must consider habitat features that do not currently exist. The modeling approach described here is flexible and can be updated for different ecosystems, species, and conservation priorities. We outline potential alterations that can be made in future analyses, depending on desired restoration goals.

## INTRODUCTION

Human activities are leading to unprecedented biodiversity declines, with land use change serving as the leading driver of biodiversity loss at a global scale (IPBES et al., 2019; Jaureguiberry et al., 2022). Conservation activities often focus on protecting wild places. While such efforts are critical for maintaining existing diversity, reversing trends in biodiversity loss will require not only conserving remaining biodiversity but also restoring degraded areas (Leclère et al., 2020; Possingham et al., 2015; Riva et al., 2024). A recent modeling exercise investigating how to stop biodiversity loss found that scenarios that halted declines and reversed trends required restoring 4.3–14.6 million km^2^ of degraded land by 2050, along with increasing the extent and management of protected areas (Leclère et al., 2020). The scenarios included in Leclère et al. (2020) may actually underestimate the effort required, since they did not consider the effects of climate change, harvest, or invasive species on biodiversity (Bryan & Archibald, 2020). In addition to increasing biodiversity, restoration can significantly increase ecosystem functioning and services (Benayas et al., 2009; IPBES, 2018). At the same time, many of these landscapes are important for livelihoods and food production, and are therefore important considerations in any restoration plans. Often, the benefits of investing in restoration far exceed the costs (IPBES, 2018).

The importance of restoration has been recognized in international, national, and regional programs. Avoiding, reducing, and reversing land degradation is necessary to achieve globally agreed upon Sustainable Development Goals (IPBES, 2018). The Convention on Biological Diversity Kunming‐Montreal Global Biodiversity Framework (GBF) includes a target that at least 30% of degraded ecosystems be under restoration by 2030 (United Nations Convention on Biological Diversity, 2022). At a national level, the United States enacted the Infrastructure Investment and Jobs Act (also called the Bipartisan Infrastructure Law, passed in 2021) which included $1.4 billion for ecosystem restoration and resilience (U.S. Department of the Interior, 2022).

Given the increasing attention to restoration, it is important to consider where investments will most effectively achieve multiple biodiversity and ecosystem services goals while minimizing costs (Future Earth and GEO BON, 2022). Conservation benefits can be measured in a variety of ways, for example by considering species of concern, overall species richness, or using benefit/utility functions (Arponen et al., 2005; Dreiss & Malcom, 2022; Hamilton et al., 2022), while costs could include acquisition and management costs (Armsworth et al., 2011) or opportunity costs from protecting and restoring land rather than using it for agriculture or other activities (Naidoo & Iwamura, 2007; Venter et al., 2014). Considering benefits and costs can allow for land management decisions that optimize conservation and economic returns (Polasky et al., 2008). Researchers have produced restoration priority maps at the global scale (e.g., Mappin et al., 2019; Strassburg et al., 2020), as well as for some regions and ecosystems in regions including the United States (e.g., Ager et al., 2013; Allan et al., 2015; Chimner et al., 2010). While global priority maps are important, many conservation decisions are made at national and regional levels, so more detailed maps are needed for effective implementation (Nelson et al., 2009).

Ecosystems that are especially degraded are good candidates for fine‐scale restoration prioritization. In some areas of North America, native prairie loss is as high as 99% since European settlement (Samson & Knopf, 1994). Tallgrass prairies have experienced the most extensive losses, but mixed‐grass and shortgrass prairie loss has also been high (Augustine et al., 2021). Grassland loss has negative consequences for biodiversity, including bird, mammal, and plant species (Laliberte & Ripple, 2004; Leach & Givnish, 1996; Morrison et al., 2007; Rosenberg et al., 2019). For example, grassland birds have exhibited the largest population declines compared to birds in any other habitat type (Rosenberg et al., 2019). Given the high rates of ecosystem degradation and biodiversity loss, restoring grasslands represents an important conservation priority.

**FIGURE 1:**
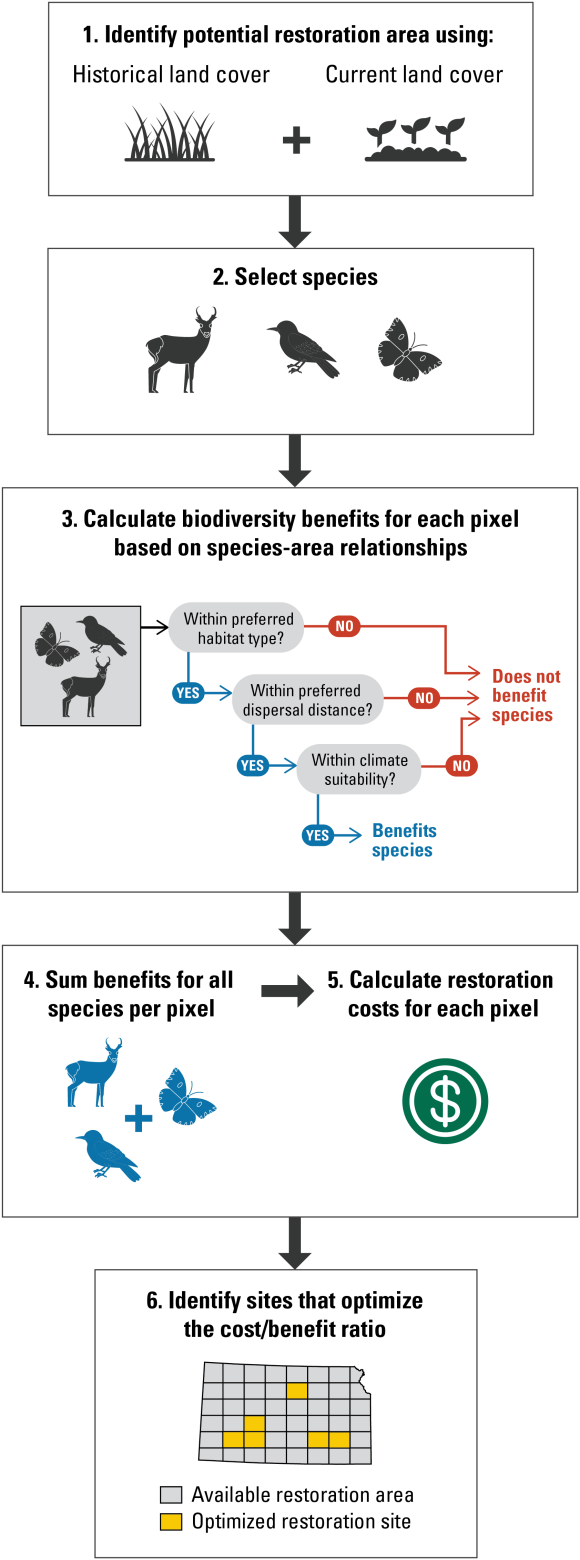
A conceptual diagram outlining the modeling approach. General steps include: (1) Consider historical and current land use to determine potential restoration area. (2) Select species of interest. (3) Assess the benefit of individual parcels on the landscape. If a parcel (i.e., pixel) is inside a species' historical range, preferred historical habitat type, and climate suitability, restoration of the pixel reduces extinction risks. Reduced extinction risk is calculated using a modified species–area relationship based on historical and current species ranges. (4) Benefits are summed across all species to calculate a total restoration benefit for that pixel. (5) Identify restoration costs for each pixel. (6) Identify sites for restoration that maximize the cost–benefit ratio using linear programming. Icons were obtained with permission from the Noun Project.

When deciding whether and where to restore land, managers may wish to know what type of ecosystem was there previously, how likely it is to support species of interest, how much restoration would cost, and how resilient it is to climate change. Here, we developed a modeling framework to maximize conservation benefit/restoration cost ratios to map restoration priorities in the United States for five obligate grassland wildlife species. By considering historical, current, and future landscape conditions and climate suitability, our framework advances standardized optimization processes. The general steps for this approach include (Figure 1):

1. Consider historical and current land use to determine potential restoration area.
2. Select species of interest.
3. Assess the benefit of restoring individual parcels on the landscape. If a parcel (i.e., pixel) overlaps a species' historical range, preferred historical habitat type, and climate suitability, restoration of the pixel reduces extinction risks. Reduced extinction risk is calculated using a modified species–area relationship based on historical and current species ranges.
4. Aggregate benefits across all species to calculate a total restoration benefit for that pixel.
5. Identify restoration costs—in this case, we used land acquisition costs, but other costs could be added if available.
6. Maximize cost–benefit ratio using linear programming.

We illustrate this framework using a case study in the Great Plains region of Kansas, USA, a recent hotspot of cropland expansion (Lark et al., 2015). We selected five grassland species that have experienced population declines from grassland loss as indicators of restoration benefit across taxa: greater prairie chicken (*Tympanuchus cupido*), lesser prairie chicken (Tympanuchus pallidicinctus), swift fox (*Vulpes velox*), pronghorn (*Antilocapra americana*), and regal fritillary (*Speyeria idalia*). We set a target of restoring 30% of land available for restoration consistent with the globally agreed GBF target (United Nations Convention on Biological Diversity, 2022). We explored how different scenarios (i.e., considering dispersal distance and future climate suitability using species distribution models [SDMs]) and model assumptions can change priorities. We expected that incorporating additional factors such as dispersal capability and climate change would increase the costs of restoration by reducing the number of places where restoration would benefit the indicator species.

## METHODS

### Land area available for restoration

We downloaded an estimated historical distribution of grassland communities of the Southern Great Plains from the U.S. Geological Survey, which provides gridded 30 × 30 m resolution estimates of 10 grassland communities prior to Euro‐American settlement (Callan et al., 2016; Reese et al., 2016). We subset to tallgrass, mixed‐grass, or short‐grass prairie in Kansas. We selected Kansas as our focal area because the state has a large amount of cropland and rangeland, is a hotspot of ongoing agricultural expansion, and was located within the historical range of our indicator species (Appendix S1: Table S1) (Lark et al., 2015). Kansas is also unique in that it contains part of the Flint Hills—the largest remaining tallgrass prairie in the United States. We restricted possible restoration sites to current cropland or pasture rather than other developed lands, as these areas are likely easier to restore than more urbanized areas, for example, and programs exist to target conservation activities on agricultural lands (e.g., the Conservation Reserve Program [CRP]). We identified current areas of cropland or pasture from the 2019 National Land Cover Dataset (NLCD; categories 81 and 82) (Dewitz and U.S. Geological Survey, 2021) and used this raster to mask the grassland community map using the raster package (Hijmans, 2020) in R version 4.0.4 (R Core Team, 2021).

### Restoration costs

Restoration costs include acquiring land, implementing restoration actions, and ultimately managing restored land. Restoration and management costs are not well documented or consistently reported; therefore, we chose to use land values for our analysis. Considering land value alone is an underestimate of overall costs. Although acquisition costs and management or restoration costs are not always correlated (Armsworth et al., 2011), estimates of land value can be used as proxies for restoration costs, since they relate to costs of acquisition or other conservation strategies like conservation easements (Nolte, 2020). Using course resolution cost proxies, such as county‐level metrics, can lead to inaccurate estimates of conservation budgets (Armsworth, 2014; Sutton et al., 2016), but high‐resolution estimates provide much better proxies (Nolte, 2020). We downloaded land values in the United States estimated from 2000 to 2019 sales data at 480 × 480 m resolution from Nolte (2020) (Appendix S1: Figure S1). We converted the cost data from ln USD/ha to USD/ha and used this dataset to estimate the cost $\left({\mathrm{Cx}}_{i}\right)$ of each pixel (*x*_*i*_). We used the “aggregate” function in terra to change the resolution of the cost layer when running models at lower resolutions (e.g., 960 × 960 m; Hijmans, 2023).

### Species included in the model

**FIGURE 2:**
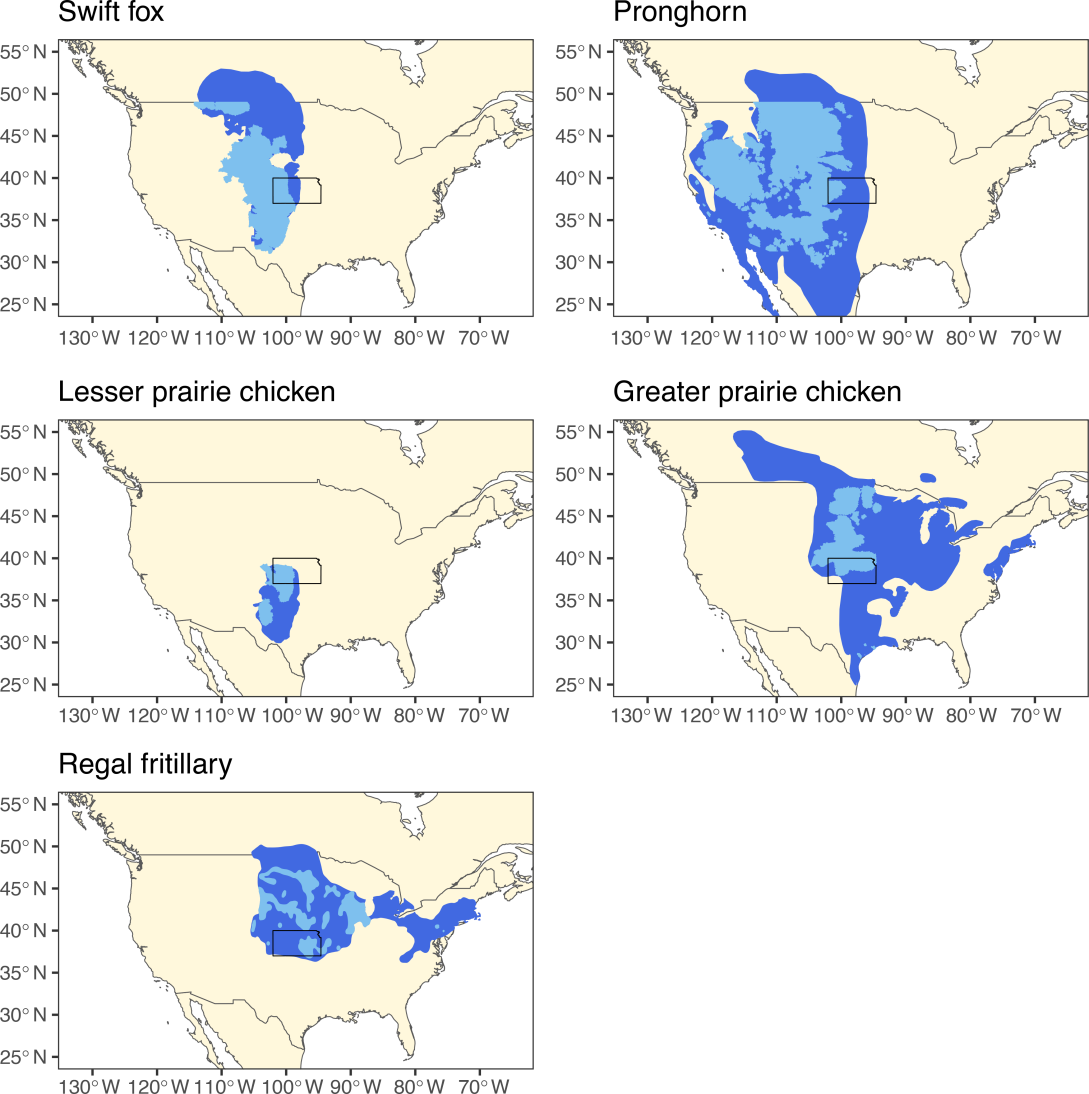
Current (light blue) and historical (dark blue) range maps for the species included in this analysis. The black box highlights Kansas, USA, in relation to the species range. The base map comes from South (2017).

We selected five grassland species as indicators of restoration benefit across taxa (current and historical range maps in Figure 2):

1. Greater prairie chickens are large grouse that have been extirpated or made extremely rare across much of their range, which only occurs in the contiguous United States and have been classified by the IUCN red list as near threatened due to loss of habitat (BirdLife International and Handbook of the Birds of the World, 2013). They are primarily found on tallgrass prairies (Robb & Schroeder, 2005).
2. Lesser prairie chickens, a smaller species found in central United States, have experienced substantial population declines from habitat loss and fragmentation (U.S. Fish and Wildlife Service, 2021). The Northern Distinct Population of lesser prairie chickens, which includes Kansas, was recently classified as threatened, while the Southern Distinct Population was classified as endangered (U.S. Fish and Wildlife Service, 2022). They are primarily found on shortgrass and mixed‐grass prairies (U.S. Fish and Wildlife Service, 2021).
3. Swift foxes, nocturnal carnivores, are found primarily on the shortgrass and mixed‐grass prairies of central United States and southern Canada. Conversion of grasslands has been one of the main drivers of the reduction in swift fox range (Moehrenschlager et al., 2004).
4. Pronghorn, the fastest land mammal, live in grassland ecosystems in western North America. Although populations have recovered, habitat loss and conversion have been drivers of population declines (IUCN SSC Antelope Specialist Group, 2016).
5. Regal fritillaries prefer mixed and tallgrass prairies and are found in central United States into southern Canada prairie (Selby, 2007). They are classified as vulnerable on the IUCN red list, with habitat loss and degradation as a primary driver of declines (Selby, 2007; Walker et al., 2022). They are restricted to habitats where their larval host plants, violets, are present (Shepherd & Debinski, 2005).

### Benefits for biodiversity

As species lose habitat, their extinction risk increases. We estimated the biodiversity benefits of restoration using a modified version of the species–area relationship to assess how restoration reduces extinction risk (Koncki & Aronson, 2015; Strassburg et al., 2020). For each species *j*, we downloaded a current range map that we used to calculate the area of current habitat ($A_{c}$) and a historical range map that we used to calculate the area of original habitat $A_{O}$ (see Appendix S1: Table S1 for sources). Based on (Strassburg et al., 2019, 2020; Thomas et al., 2004), we calculated the current extinction risk $\left(r_{0}\right)$ for each species (*j*):

(1) $$r_{0j}=1-{\left(\frac{A_{c}}{A_{O}}\right)}^{z}$$

To create a restoration benefit map for each species, we estimated the new extinction risk $\left(r_{1j}\right)$ if a particular pixel *x*_*i*_ was restored. We assumed that areas restored to each species' preferred habitat type (Appendix S1: Table S1) would eventually become habitat for the species in the long term and reduce extinction risk. Thus, if *x*_*i*_ is within the species preferred habitat type, the new extinction risk is

(2) $$r_{1j}=1-{\left(\frac{A_{c}+Ax_{i}}{A_{O}}\right)}^{z},$$

where $Ax_{i}$ is the area of pixel *x*_*i*_, and *z* is a constant representing how extinction risk scales with habitat loss. Although *z* = 0.25 is commonly used for species area relationships, we used a range of *z* = 0.1–0.4 to assess the sensitivity of our priority maps.

We then calculated the restoration benefit of each individual pixel $b_{\mathrm{ij}}$ for each species as the reduction in extinction risk achieved by restoring a particular pixel multiplied by the current or future climate suitability ($c_{\mathrm{ij}}$; see *Climate change scenarios*). Additional weights can be added to the benefit calculation as desired (e.g., see *Dispersal scenario*). This approach assumes that everything within the boundaries of the historical range map and prairie type, and within suitable climate conditions and dispersal distances (see below), could potentially be suitable for the species.

(3) $$b_{\mathrm{ij}}=c_{\mathrm{ij}}\times \left(r_{0j}-r_{1j}\right).$$

To create an overall benefit map considering all species (*S*), we calculated the restoration benefit of each pixel $\left({\mathrm{Bx}}_{i}\right)$ by summing the benefit for each individual species per pixel:

(4) $${\mathrm{Bx}}_{i}=\sum_{j=1}^{S}b_{\mathrm{ij}}.$$

### Optimization model

Multiple optimization approaches and algorithms exist that could be used to identify restoration priorities, such as Marxan, Zonation, and integer linear programming (ILP) (Ball et al., 2009; Moilanen, 2007; Moilanen et al., 2005; Watts et al., 2009). We used ILP in the R package lpSolve to optimize restoration scenarios (Berkelaar, 2023). ILP maximizes or minimizes an objective function subject to a set of constraints (Beyer et al., 2016). When objectives can be converted into linear format, ILP will find an optimal solution given the constraints and can require less computational time than other approaches, which is a benefit over other heuristic methods like simulated annealing (e.g., as used in Marxan) (Beyer et al., 2016). ILP is also flexible. It is straightforward to add additional constraints or change the structure of the analysis. For example, in our current approach, we seek to maximize the cost–benefit ratio. However, the cost layer could be moved from the maximization equation to a constraint equation to look to maximize benefits given a cost cap.

We set our objective function to maximize the benefit/cost ratio subject to a total limit on area (in this case, set to 30% of the pixels available for restoration) and a minimum amount of each habitat type to be restored.

(5) $$\max\sum_{i=1}^{N}\frac{{\mathrm{Bx}}_{i}}{{\mathrm{Cx}}_{i}},$$

subject to:

$$\sum_{i=1}^{N}Ax_{i}\leq T_{A},$$

$$\sum_{i\in G_{a}}Ax_{i}\geq H_{A},$$

where *N* = the total number of pixels available for analysis and *T*_*A*_ is the total area that can be restored in each model run. The second constraint sets a minimum area (*H*_*A*_) that needs to be restored for each habitat type (*a*), and *G*_*a*_ represents the membership of each pixel within a habitat type. To account for diminishing returns in species benefit as more suitable habitat is restored, we ran our optimization in three iterations. To do this, we set $T_{A}$ = 0.1 × *N* (i.e., after three iterations, we will have reached our 30% restoration target). We also ran the optimization with 30 iterations but obtained similar results (Appendix S1: Figure S2); we present only results from three iterations in the main text. We set $H_{A}$ = 0.067 × *G*_*a*_ (i.e., after three iterations we will restore at least 20% of each grassland habitat type). We also tested a 0% and 10% minimum threshold. After each iteration, we added newly restored sites to the current area of habitat ($A_{c}$) for each species *j* as applicable and repeated Equations ((1), (2), (3), (4), (5)) until total planned area was accounted for.

### Dispersal scenarios

Not all restored areas are equally likely to be colonized naturally. We assessed how weighting biodiversity benefits by distance to current habitat could affect restoration outcomes. We ran three scenarios that considered pixels outside of the dispersal distance of each species to have equal, partial, or no benefit:

1. No dispersal consideration scenario—All suitable habitat types were considered equally beneficial regardless of distance to current habitat (i.e., benefits calculated as described in the benefits to biodiversity section above).
2. Intermediate scenario—Pixels whose distance was within the known dispersal distance of the species current range were weighted as 1 (i.e., $b_{\mathrm{ij}}=r_{0j}-r_{1j}$), whereas those outside were given 0.5 weight (i.e., $b_{\mathrm{ij}}=0.5\times \left(r_{0j}-r_{1j}\right)$). To do this, we added a buffer equal to the dispersal distance (Appendix S1: Table S1) around the current range map. We turned this shapefile into a raster such that areas inside the buffer were equal to 1, while pixels outside were equal to 0.5, and multiplied this by the restoration benefit of each pixel $b_{\mathrm{ij}}$ for each species. This assumes that either some translocation is possible, or that eventually species will move to further habitats, but that this may take longer and thus has lower immediate benefits than closer pixels;
3. Natural dispersal scenario—Only pixels within the dispersal distance were considered beneficial, while pixels outside of this distance were given a benefit of 0. Methods were the same as the intermediate scenario, except that pixels outside the dispersal distance were set to 0.

We assessed sensitivity to pixel size by varying the resolution between 480 × 480 m (the original resolution of the cost map) and 960 × 960 m. We found that pixel size only slightly influenced the area of each habitat type selected or reduced extinction risk for each species (Appendix S1: Figure S3), so we present results for 960 × 960 m for computational efficiency.

### Climate change scenarios

Restoration may be most effective in the long term when we preferentially restore habitats that are likely to remain suitable under climate change. SDMs can be useful for restoration because they highlight places that could be suitable, even when the species is not currently present (Araújo & Peterson, 2012). We fit SDMs for each of our species under two climate change scenarios: SSP1‐2.6, a low climate change, low land use change scenario and SSP3‐7.0, a medium–high reference scenario (Meinshausen et al., 2020; van Vuuren et al., 2017). Our goal with the SDMs was to identify areas that we expect to remain climatically suitable for the species. We only included climate variables in the SDMs because other habitat requirements (e.g., grassland type) are incorporated in the restoration optimization model itself and not the SDM. This is because the current landscape likely does not contain the land use or vegetation structure required by the species but could be restored to those conditions.

#### Biodiversity data

We downloaded species presence data from the Global Biodiversity Information Facility (GBIF) Darwin Core Archive (GBIF.org, 2024). We excluded fossil and living specimens, observations without coordinates, and observations with reported coordinate uncertainty larger than 5 km or less than 3 m (i.e., those with very large uncertainty or that are suspiciously precise). We used the coordinate cleaner R package to exclude coordinates that had equal latitude and longitude, zero coordinates, or marine coordinates and those located at country capitals, country centroids, biodiversity institutions, or GBIF headquarters (Zizka et al., 2019). We also excluded any points that were more than 10 km outside of their current or historical range maps. We thinned coordinates so that we had only one observation per climate raster cell to reduce clumping and sampling bias (Boria et al., 2014; Soley‐Guardia et al., 2024).

#### Climate data

We downloaded 30‐year average climatologies at a 30 arc‐sec resolution from CHELSA (Karger, Conrad, et al., 2021; Karger et al., 2017) for mean daily maximum air temperature of the warmest month, mean daily minimum air temperature of the coldest month, precipitation of the wettest month, and precipitation of the driest month. We used data from 1981 to 2010 to fit the model and projection data for 2041–2070 for future suitability projections. For the projections, we used the CHELSA downscaled outputs from the CMIP6 GFDL‐ESM4 climate model (Karger, Brun, & Zimmermann, 2021). We tested for collinearity and excluded variables with greater than ±0.8. Final variables included in the model varied by species, and we considered species biology in final selection (see Appendix S1: Table S2 for details and justifications). We clipped climate layers to 70–130° W and 25–55° N to cover the historical range of each species.

#### Elevation data

We used the “terrain” function in terra (Hijmans, 2023) to calculate slope from the 7.5‐arc second resolution USGS global multi‐resolution terrain elevation data 2010 (GMTED2010; Danielson & Gesch, 2011). We resampled the data to match the resolution of the climate data using the “resample” function in terra (Hijmans, 2023).

#### Species distribution models (SDMs)

We fit two types of SDMs for each species—MaxEnt and random forest. We reprojected all spatial data to an equal area projection—NAD83 / Conus Albers (EPSG:5070) (Elith et al., 2011). We fit MaxEnt models using the maxent function in the dismo package using default model settings (Hijmans et al., 2023; Phillips et al., 2006). To test model performance, we used 5‐fold cross‐validation and took the average area under the receiver–operator curve (AUC) for each species. For model validation, we randomly generated 1000 background points. For the final model, we used all species data.

We fit SDMs using the randomForest function in the randomForest package (Liaw & Wiener, 2002). We generated 10,000 random background points and extracted climate data for presence and background points. We ran 1000 classification trees using downsampling random forest as recommended by (Valavi et al., 2021) to account for the imbalanced number of presence and background points. That is, each classification tree used the same number of background samples as presence samples by sampling from the background points with replacement. Again, we tested model performance using 5‐fold cross‐validation, took the average AUC for each species, and used all occurrence data in the final model.

For all SDMs, we fit and tested the model with historical climate data. We then predicted habitat suitability using future climate projections using the original modeling area but clipped the results to Kansas for use in the restoration model. We calculated multivariate environmental similarity surfaces for both climate scenarios using the “mess” function in the dismo (Appendix S1: Figure S4) (Elith et al., 2010; Hijmans et al., 2023). See Appendix S2 for our complete ODMAP (Overview, Data, Model, Assessment and Prediction) protocol checklist (Zurell et al., 2020).

#### Inclusion in restoration model

To incorporate climate into the restoration scenarios, we multiplied the SDM suitability estimates by the restoration benefit of each pixel for each species ($c_{\mathrm{ij}}$ in Equation 3). Because differences were small between SSP1‐2.6 and SSP3‐7.0, we present the results for SSP3‐7.0 in the main text. For models of current condition, we present results using the MaxEnt models with historical climate data.

## RESULTS

**FIGURE 3:**
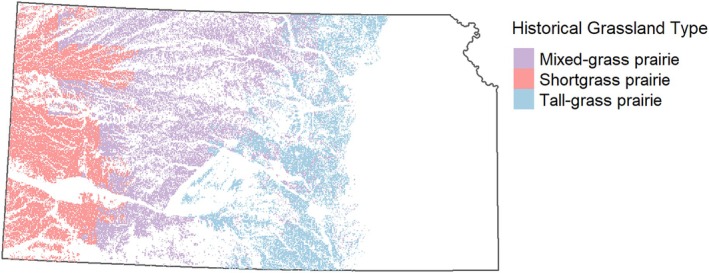
Area in Kansas, USA, that is, currently cropland or pasture by historical grassland community type. This was considered the area available for restoration for this analysis.

Available restoration area ranged from 21,874 km^2^ of shortgrass prairie in western Kansas to 29,311 km^2^ of mixed‐grass prairie in central Kansas to 16,656 km^2^ of tallgrass prairie in eastern Kansas, comprising about 32% of the state (Figure 3). Restoring 30% of this available area with at least 20% of each habitat type could cost on the order of $5 billion, averaging ∼$2500/ha (Table 1) across all indicator species and scenarios.

**TABLE 1:** Total restoration costs for each restoration scenario (rounded to the nearest $10) run at 960 × 960 m resolution and a 20% minimum habitat restoration for each habitat type.

Scenario	Total cost	Average cost/ha
No dispersal consideration	$4,951,131,120	$2430
Intermediate	$5,141,658,750	$2530
Natural dispersal	$5,297,537,120	$2600
Maxent SSP3‐7.0	$4,930,020,570	$2420
RF SSP3‐7.0	$4,992,845,050	$2450

*Note*: Scenarios were run using current cropland and pasture as potential restoration area. Species assessed included swift fox, pronghorn, lesser prairie chicken, greater prairie chicken, and regal fritillary.

### Dispersal and climate scenarios

**FIGURE 4:**
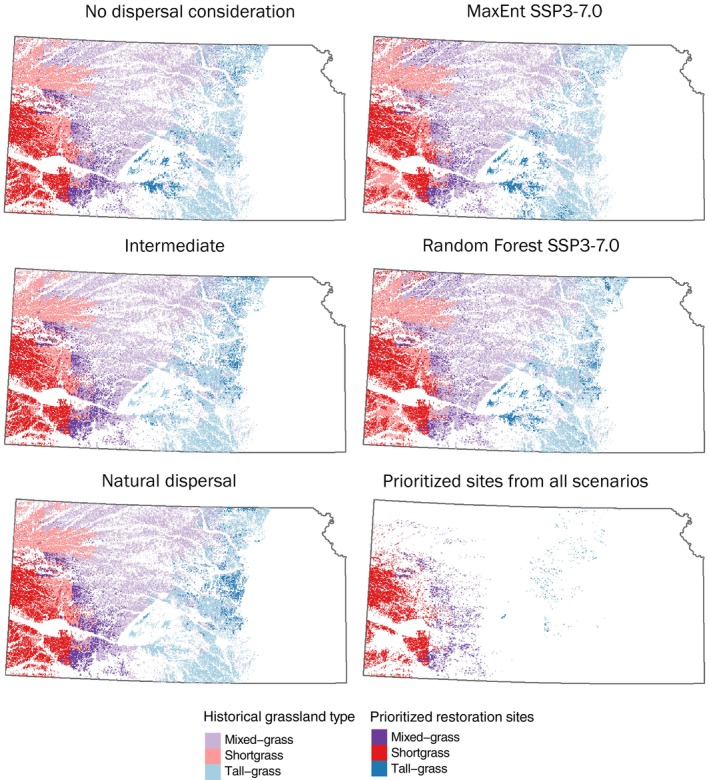
Selected restoration sites for the dispersal and climate change scenarios, as well as a map of sites that were selected across all five scenarios (bottom right). Results are from scenarios where *z* = 0.25, minimum habitat area to be restored per habitat type is 20%, resolution = 960 × 960 m, iterations = 3.

As dispersal limitations became more influential in selecting priority restoration sites (i.e., moving from the no dispersal consideration scenario to the natural dispersal scenario), northern areas of tallgrass prairie habitat were selected while mixed‐grass and shortgrass prairie sites were similar (Figure 4). Priority maps were similar when climate change scenarios were considered, but MaxEnt models selected western tallgrass prairie locations while random forest models selected tallgrass prairie farther north (Figure 4, Appendix S1: Figure S5). There were areas that were selected in all scenarios (Figure 4).

**FIGURE 5:**
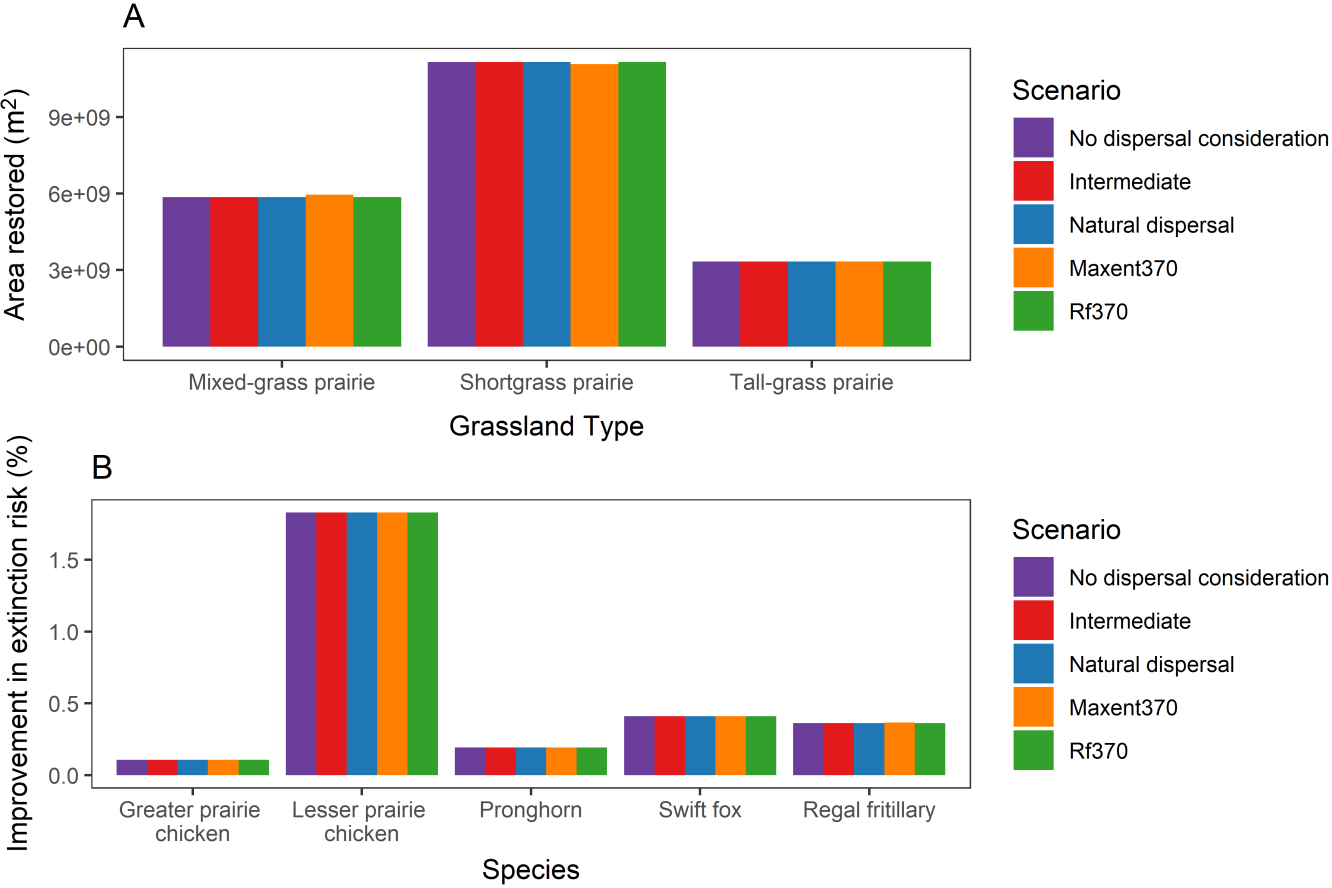
Area restored in each habitat type (A) and improved extinction risk for each species (B) under dispersal and climate change restoration scenarios. Results are from scenarios where *z* = 0.25, minimum habitat area to be restored per habitat type is 20%, resolution = 960 × 960 m, iterations = 3.

Although restoration location shifted, total area restored in each habitat type was similar under each scenario (Figure 5, Appendix S1: Table S3). Lesser prairie chicken had the greatest reduction in extinction risk (0.86%–2.5% when we set a 20% minimum habitat restoration threshold under the full range of values we considered for how extinction risk scales with habitat loss), while greater prairie chickens had the least (0.06%–0.13%; Appendix S1: Table S4).

The natural dispersal scenario was the most expensive, followed by the intermediate scenario, then the no dispersal consideration and climate scenarios (Table 1). The natural dispersal scenario was more expensive because the restriction on distance to current habitat necessitated the selection of more expensive places. Even so, the natural dispersal scenario was only ∼7% more expensive than the no dispersal consideration scenario.

### Minimum habitat threshold scenarios

**FIGURE 6:**
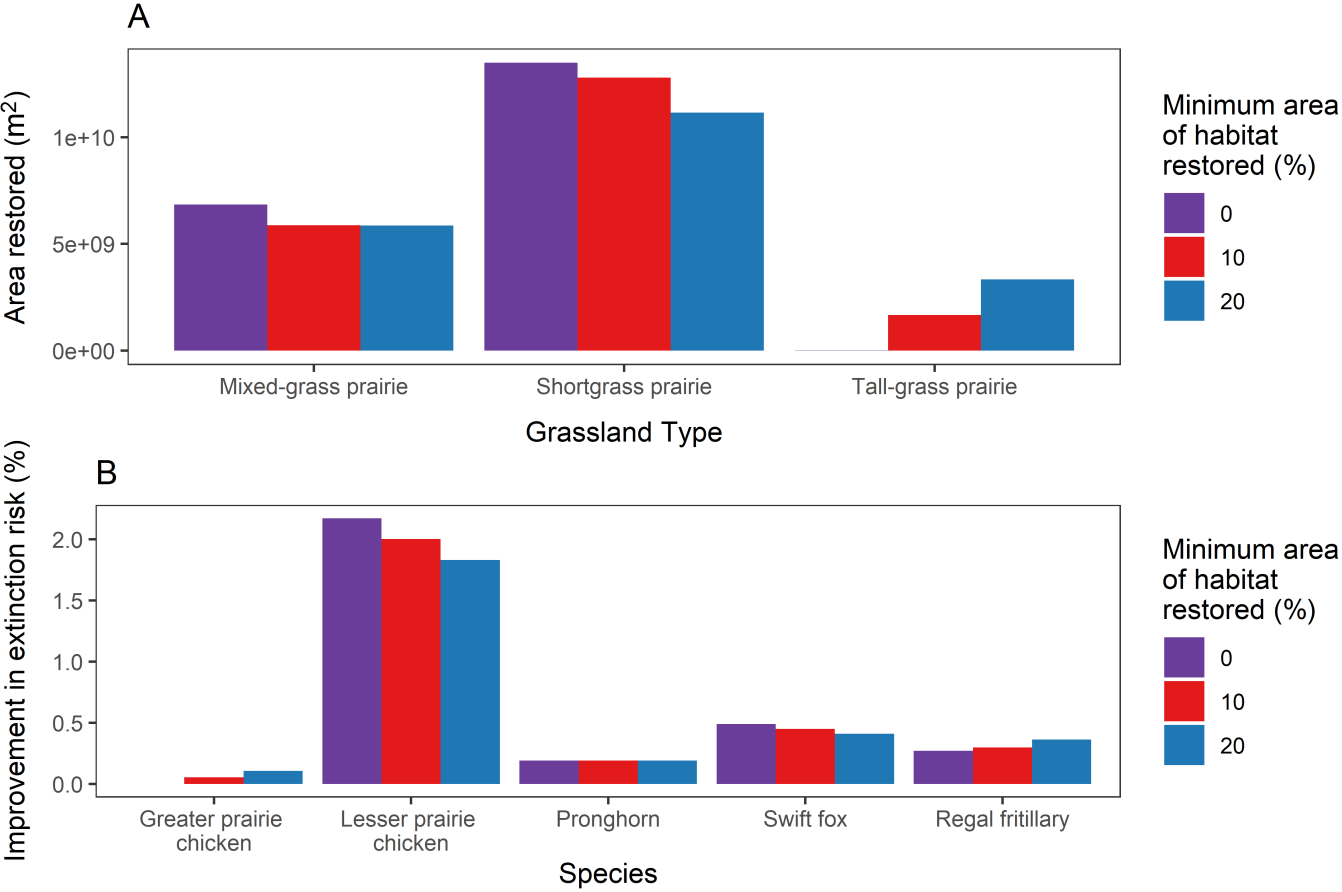
Comparison of how the minimum area of each habitat type restored affected area restored by grassland type (A) and improved species' extinction risk (B) under the no dispersal consideration scenario (i.e., all suitable habitat types were considered equally beneficial for the species regardless of distance to current habitat).

When we set a lower threshold for minimum area of habitat restored for each habitat type, we saw similar patterns, but less tallgrass prairie was selected (Appendix S1: Figure S6, Figure 6). Setting a higher minimum habitat threshold reduced extinction risk slightly for the tallgrass prairie species (greater prairie chicken and regal fritillary), but increased extinction risk for species that do not use tallgrass prairie (lesser prairie chicken and swift fox). Cost differences were negligible between scenarios (Table 2).

**TABLE 2:** Total restoration costs for different minimum habitat thresholds for the no dispersal consideration scenario at 960 × 960 m resolution.

Minimum habitat scenario	Total cost	Average cost/ha
0%	$4,955,565,350	$2430
10%	$4,905,992,800	$2410
20%	$4,951,131,120	$2430

*Note*: Scenarios were run using current cropland and pasture as potential restoration area. Species assessed included swift fox, pronghorn, lesser prairie chicken, greater prairie chicken, and regal fritillary.

## DISCUSSION

Ecological restoration will be important for conservation as countries work to achieve the global biodiversity targets in the GBF. We developed a modeling approach to prioritize restoration actions and illustrated the model's utility using a case study in Kansas. We found that, for the indicator species that we chose, shortgrass and mixed‐grass prairies had the highest conservation benefit to cost ratio. However, restoration costs, even considering only acquisition costs, were extremely high to restore 30% of the land area and may not be a feasible target. Setting a minimum restoration threshold for each habitat type allowed us to identify high‐priority tallgrass prairie sites. Thresholds and species can be altered in future scenarios to reflect management needs.

Prioritizing restoration is challenging because data on potential habitat features do not currently exist (Yoshioka et al., 2014). Previous approaches have addressed this challenge by using optimization algorithms like Marxan and Zonation to minimize costs while restoring a certain percentage of habitat cover or simulating fragmentation effects from a hypothetical fully vegetated landscape (Crouzeilles et al., 2015; Thomson et al., 2009). We used an approach similar to Strassburg et al. (Strassburg et al., 2020), where we used species–area relationships to estimate restoration benefits for biodiversity. This approach allowed us to incorporate biodiversity benefits into the optimization and account for diminishing returns as more habitat is restored. Strassburg et al. (2020) ran their model globally and used over 20,000 species. Running the analysis on a smaller scale allowed us to consider more detailed historical habitat and estimated historical ranges of our modeled species. Moreover, when considering return on investment globally, higher income countries like the United States may not come out as the best locations for restoration (Luby et al., 2022). However, there is interest and funding for restoration within the United States. It is therefore worthwhile to assess restoration priorities nationally and within ecosystems of concern such as grasslands.

Altering the number of iterations, weighting benefits based on dispersal distance and climate scenarios, and using different pixel sizes and species–area relationships did not greatly change the prioritization. There were locations that were selected under all scenarios. These locations might be higher priorities because they are robust to multiple model assumptions. Considering differential benefits based on dispersal distance and current location is useful, as the current species range may more closely reflect the species' niche than its historical range (Lenoir et al., 2020; Rubenstein et al., 2023). In addition to considering current species locations, we considered possible future suitability by using SDMs and multiple climate change scenarios. Considering climate change is important because in some cases achieving historical baselines will no longer be feasible (Temperton et al., 2019). Climate change had the greatest effect on regal fritillary suitability as Kansas is currently located at the southern edge of the species range (Figure 2, Appendix S1: Figure S7). Climate is not the only source of non‐stationarity; non‐stationarity can also arise from altered species interactions, habitat associations, and management contexts (Lawler et al., 2010; Nichols et al., 2011; Post van der Burg, 2024). We attempted to incorporate some biological non‐stationarity by using a range of values for how species extinction risk scales with habitat loss.

Our restoration prioritization approach could add value to existing planning and conservation initiatives. For example, Comer et al. (2018) identified potential conservation areas in the Great Plains (including Kansas), and Werdel et al. (2023) identified priority native grassland sites for swift foxes. Our priority tallgrass, mixed‐grass, and shortgrass prairie selections could be overlaid with the potential conservation areas to further prioritize restoration sites.

While we found that the no dispersal consideration scenario was the least expensive (Table 1), this scenario might cost more if intensive translocation efforts are required to restore species. Restoration cost reporting is limited and inconsistent, resulting in huge variation in estimated restoration costs (Iacona et al., 2018; Knight & Overbeck, 2021). For example, a survey of practitioners found that grassland restoration costs varied from USD 13/ha to 79,255/ha (Knight & Overbeck, 2021). Land use intensity and level of degradation affect project costs and success. Throughout the Great Plains, tallgrass prairies are typically more degraded and thus likely more difficult to restore than shortgrass and mixed‐grass prairie (Comer et al., 2018). Kansas may be the exception, since it contains one of the largest remaining tallgrass prairies (With et al., 2008). Future work that improves our understanding of restoration costs would improve the cost estimates in our analysis.

For our cost estimation, we used acquisition costs, but acquisition and management or restoration costs are not always correlated (Armsworth et al., 2011). Funding will be needed not just to acquire land, but also to implement restoration actions and manage the land in the long term. One challenge for restoration in the Great Plains is that much of the land is privately owned (Augustine et al., 2021). It is therefore unlikely that all of the selected land parcels would be purchased outright, especially because landowners depend on the land for their livelihoods. There are programs to promote conservation and restoration on agricultural lands that do not require land purchases. For example, the CRP allows agricultural producers to voluntarily take land out of production and improve it for conservation in exchange for rental payments (USDA Farm Service Agency, 2022). However, conservation needs far exceed available restoration budgets, and many landowners are unable to re‐enroll their expiring CRP lands even when they want to (Barnes et al., 2020). Insufficient financial or informational/technical support can lead farmers to stop long‐term grassland management (Waldén & Lindborg, 2018). For example, high crop prices can also incentivize farmers not to renew CRP agreements and turn grasslands back into crop production (Wright & Wimberly, 2013).

Efficiently choosing restoration sites, as well as developing more biodiverse working landscapes and partnerships with landowners may be needed to meet conservation goals and may be more sustainable in the long term (Augustine et al., 2021). An example is the Natural Resources Conservation Service Working Lands for Wildlife (WLFW) program, a voluntary, incentive‐based program that aims to improve wildlife habitat on working lands (Natural Resources Conservation Service (NRCS), 2021). The WLFW program can complement the CRP by replacing lost CRP revenue with sustainable livestock grazing, thus keeping the land as grassland rather than converting back to agriculture (Natural Resources Conservation Service [NRCS], 2021).

Even as croplands are pulled out of production and restored through programs like the CRP, additional grasslands continue to be converted. Over a quarter of converted grasslands between 2008 and 2012 came from long‐standing prairie locations (Lark et al., 2015). Additionally, CRP restoration may not be as beneficial for shortgrass prairie species like swift foxes because the seeds used in the restoration often result in taller vegetation (Werdel et al., 2022). Thus, preventing the conversion of intact grasslands is also important.

Our model assumes that restored pixels will eventually become habitat for our indicator species. While we used dispersal distance as a weighting factor to calculate benefits for the natural dispersal and intermediate scenarios, we still included the final habitat selection in the extinction risk calculation. The species–area relationship considers long‐term (i.e., in the coming decades), not near‐term, extinction risk and ultimately these places may become habitat for the species and contribute to the species' survival. However, many factors affect restoration success, and definitions of success or what constitutes a biodiverse restoration can vary (Andres et al., 2022). For example, it is much easier and less expensive to restore a subset of plants back on the landscape than to fully restore native plant diversity (Martin et al., 2005). Our final optimized scenarios also assume that restoration could be implemented all at once, which is not realistic. The natural dispersal scenario, in particular, assumes that restoration is occurring within one generation of the species (i.e., benefits are only considered if the site is within the dispersal distance of the current habitat). Our model was run iteratively, such that optimized areas were selected sequentially based on the cost–benefit ratio in that specific run. This approach can allow for sequential implementation of restoration by setting the desired amount of habitat area to be restored in each run, and then looking at the sequential output of optimized areas. This may be useful to identify the highest priority land areas, which is important given the high costs of purchasing and restoring all land area at once. Such an approach can allow for smaller, targeted pilot projects that can be expanded as funding allows. Our optimized scenarios cost ∼$2500 per ha, thus smaller scale restoration projects may have manageable costs. Additionally, the model could be revised to maximize benefits for a given budget (see *Methods: Optimization model*), thus allowing managers to optimize restoration areas given their available budgets.

### Future model applications

Our modeling approach can be modified or scaled up depending on management needs. For example, indicator species could be adjusted depending on the target ecosystem and management goals. Model outcomes are highly influenced by which species are included in the model, and there can be trade‐offs between the best conservation outcomes for different species. Managing trade‐offs and deciding which species to prioritize depend on the values of decision‐makers and stakeholders, and may need to be decided through negotiations or a structured decision‐making process. Following these discussions, species can be weighted in the model so that some species benefits are more influential in optimization model. That is, Equation (4) could be modified to ${\mathrm{Bx}}_{i}=\sum_{j=1}^{S}w_{j}\times b_{\mathrm{ij}}$, where *w* = the weight given to each species. We only included species for which we have an estimated historical range. Other approaches could include estimating historical ranges using preferred habitat types, species distribution models, or expert opinions.

In addition to considering land acquisition costs, managers may also want to consider restoration feasibility. Restoration feasibility depends on the level of degradation and the amount of time since land conversion. A recently converted agricultural site may still have a native seedbank, whereas places with a longer history of cultivation may require seeding (Bakker & Berendse, 1999; Wang et al., 2017). Seeds for some prairie species are difficult to collect, which can make it challenging to include all remnant species in seed mixes (Newbold et al., 2020). One near‐term next step would be to use a measure of land use intensity as a proxy of restoration effort (e.g., Suraci et al., 2023). This assumes that more intensively used landscapes are more difficult to restore.

Our model could be modified to include other ecosystem services. For example, grassland restoration can have large benefits for carbon storage and for cultural services like access to nature (Bengtsson et al., 2019; Samson & Knopf, 1994). Global analyses have incorporated carbon storage as an additional restoration benefit (e.g., Strassburg et al., 2020). Such an approach may allow for additional sources of restoration funding, such as carbon credits. In the future, it may be possible to add other metrics like distance to population centers to approximate access to native ecosystems.

Finally, users may wish to account for other aspects of landscape configuration. For example, Henslow's sparrow (a grassland specialist; *Ammodramus henslowii*) populations increased more in areas with high local CRP enrollment (Herkert, 2007). Depending on conservation goals, clusters of restored pixels may be more beneficial than a single restored pixel, and weighting could be added to the model to reflect this. Formal connectivity analysis could also be conducted for species in the model and used as a weight on the benefit layer (i.e., make the dispersal scenarios more complex).

## CONCLUSION

Given limited budgets, deciding where to restore habitat will be an important need in the coming decade. We developed a modeling approach that can be used to maximize conservation benefit to cost ratios. Optimizing restoration is challenging because one must consider habitat features that do not currently exist. We illustrate how to do this using historical and current landscape conditions and species ranges while also considering current and future climate suitability. Our approach is flexible and can be updated for different ecosystems, species, and conservation priorities. We also outlined potential alterations that can be made in future analyses, depending on desired restoration goals.

Global and domestic conservation targets highlight the growing recognition that ecological restoration will be needed to meet biodiversity conservation goals. Ecological restoration improves biodiversity and ecosystem services compared to degraded landscapes, but restored areas remain consistently less diverse and provide fewer benefits than unaltered reference ecosystems (Benayas et al., 2009; Martin & Wilsey, 2006; Newbold et al., 2020). Improving restoration techniques will thus be a critical challenge in the coming years (Brancalion & Holl, 2025), especially for ecosystems like grasslands that have little remaining intact habitat.

## AUTHOR CONTRIBUTIONS

Sarah R. Weiskopf, Susannah B. Lerman, and Toni Lyn Morelli designed the study. Sarah R. Weiskopf collected data. Sarah R. Weiskopf and Alexey N. Shiklomanov analyzed data. Sarah R. Weiskopf led the writing of the manuscript. All authors contributed substantially to revisions.

## CONFLICT OF INTEREST STATEMENT

The authors declare no conflicts of interest.

## Supporting information


Appendix S1.

Appendix S2.

## Data Availability

Data (Weiskopf et al., 2025) are available in the USGS ScienceBase repository at https://doi.org/10.5066/P139JZZK. Code is published in a USGS code release available at https://doi.org/10.5066/P14TDJUR.
