# Supplementary material for: Restoration based on cost–benefit optimization: A grasslands pilot study
Source: Ecol Appl. 2026 Jan 29;36(1):e70174. doi: 10.1002/eap.70174 (PMC12854812; doi:10.1002/eap.70174)
Supplement: Supplementary file 1 — Appendix S1. [file EAP-36-e70174-s001.pdf]

Appendix S1

Restoration based on cost–benefit optimization: A grasslands pilot study

Sarah R. Weiskopf, Toni Lyn Morelli, Tina G. Mozelewski, Alexey N. Shiklomanov, Susannah B. Lerman

Ecological Applications

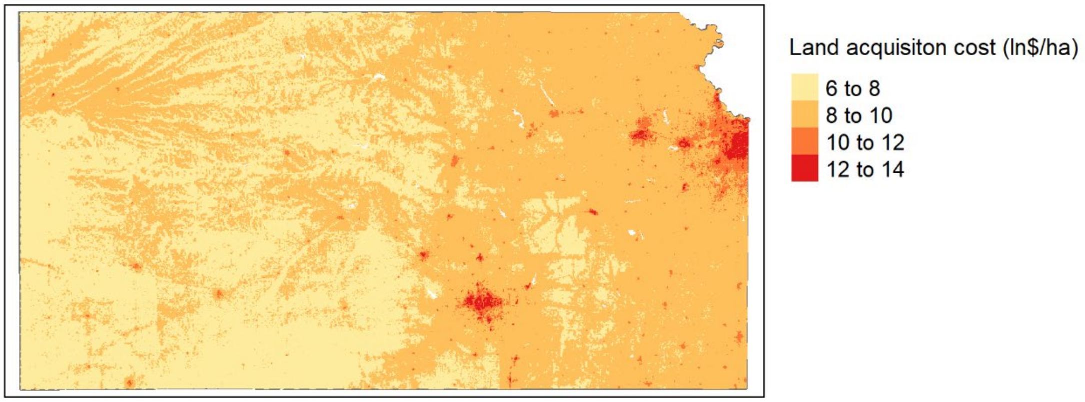

Figure S1: Land acquisition costs across the study area in ln$/ha. Data comes from (Nolte 2020)

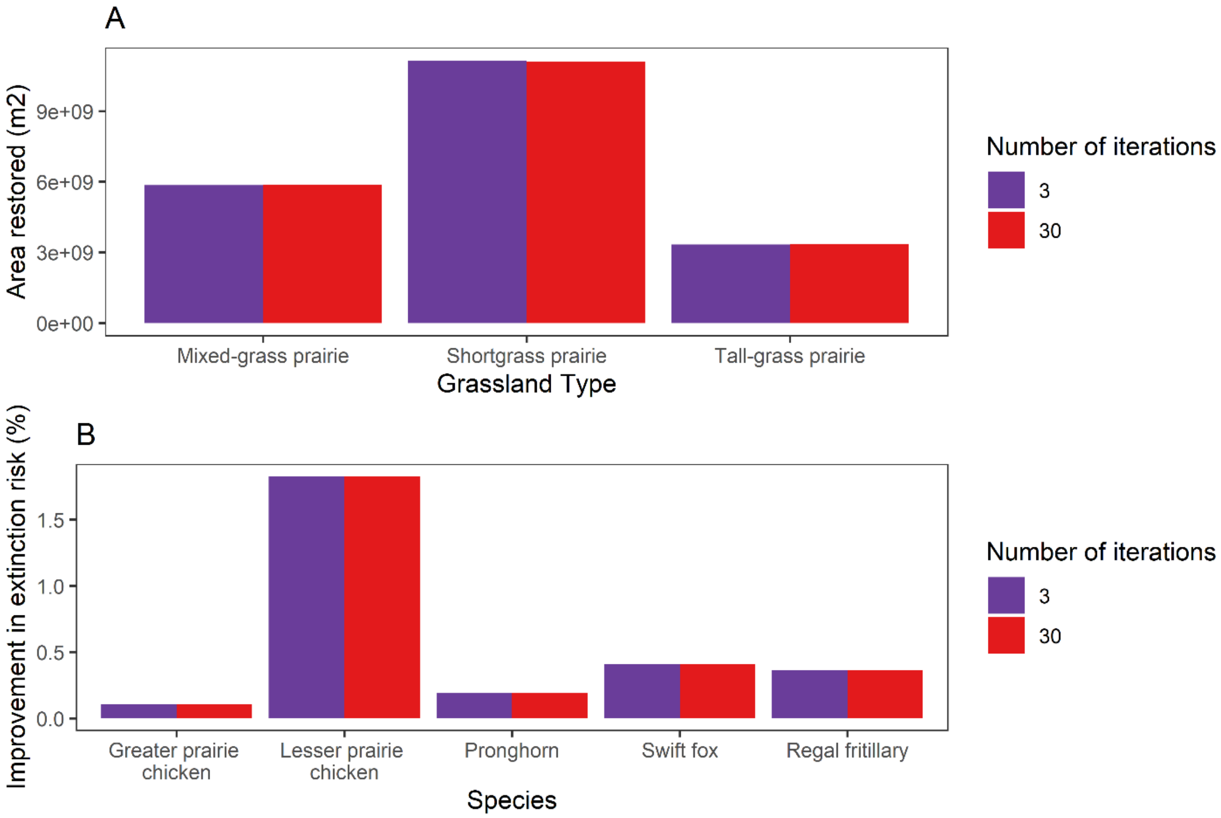

Figure S2: Comparison of how the number of iterations used in the optimization affected area restored by grassland type (A) and improved species' extinction risk (B) under the translocation scenario (i.e., all suitable habitat types were considered equally beneficial for the species regardless of distance to current habitat) and using a 20% minimum restoration threshold per habitat type.

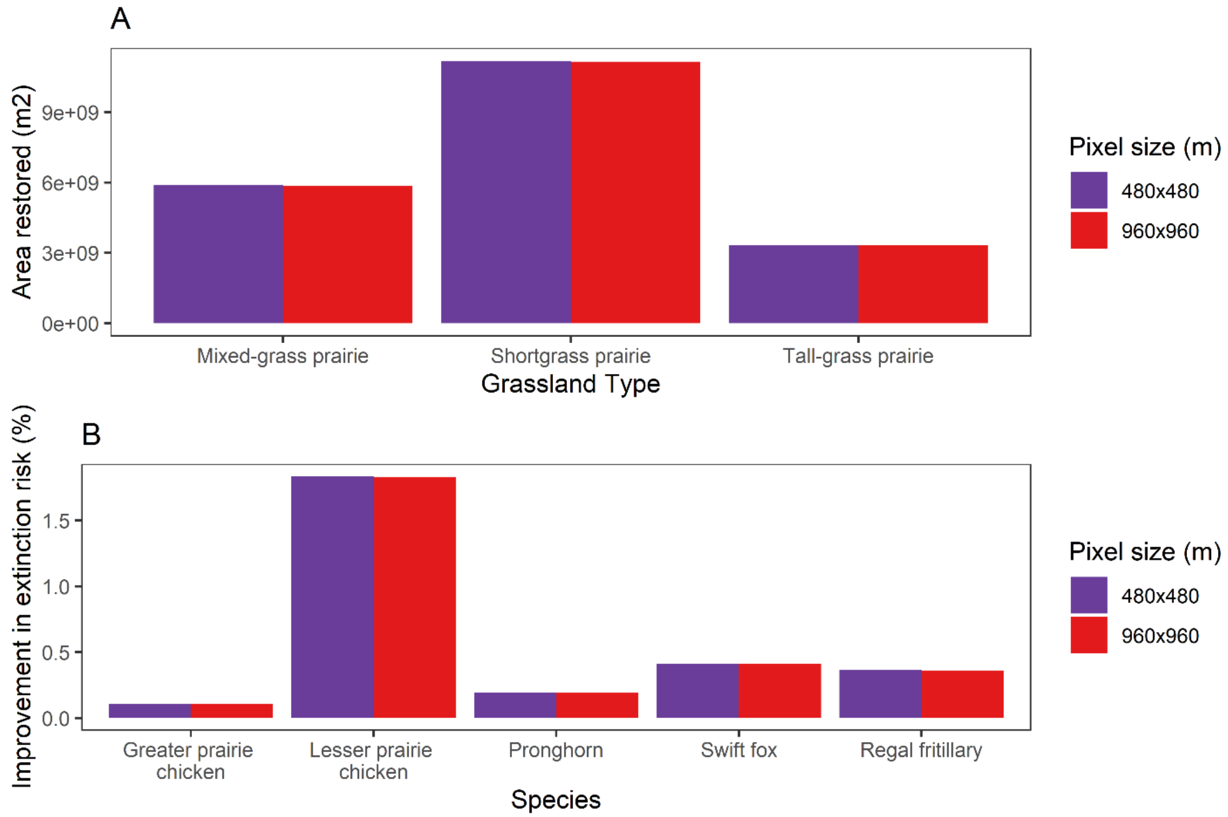

Figure S3: Comparison of how pixel size (resolution) affected area restored by grassland type (A) and improved species' extinction risk (B) under the translocation scenario (i.e., all suitable habitat types were considered equally beneficial for the species regardless of distance to current habitat) and using a 20% minimum restoration threshold per habitat type.

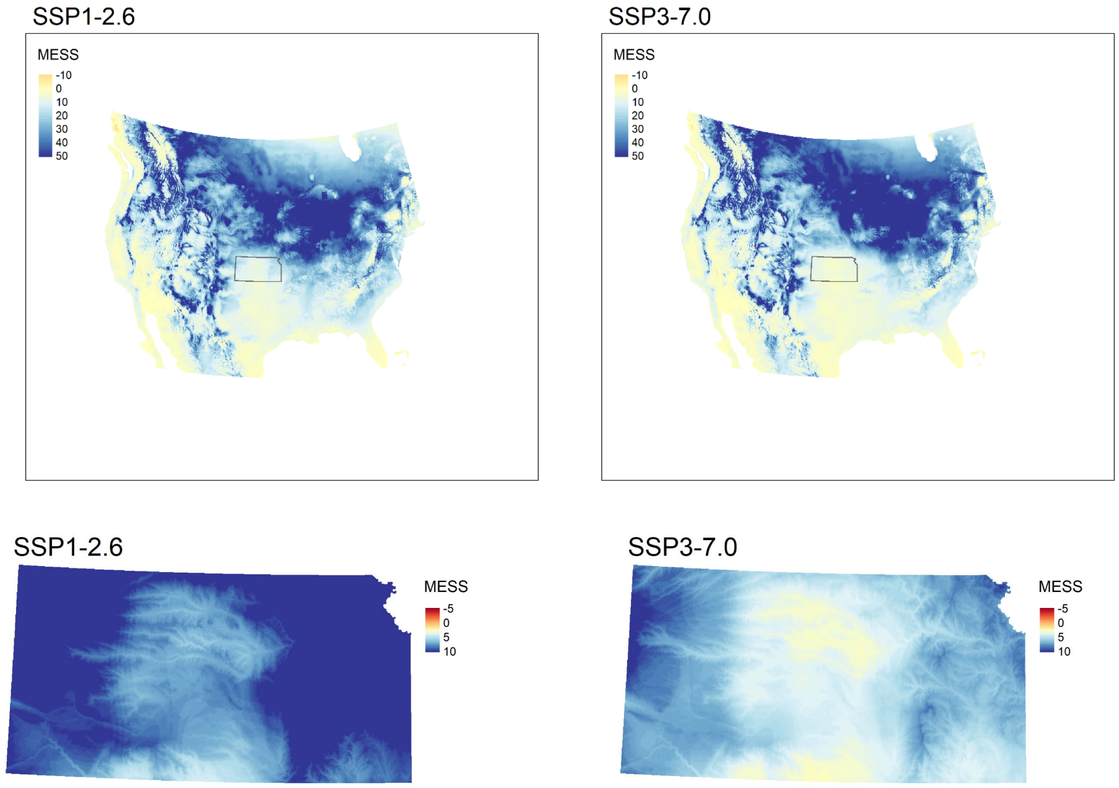

Figure S4: Multivariate environmental similarity surface (MESS) under SSP1-2.6 and SSP3-7.0 for the entire modeling area and subset to Kansas. Negative numbers indicate a novel environment.

Maxent SSP 1-2.6

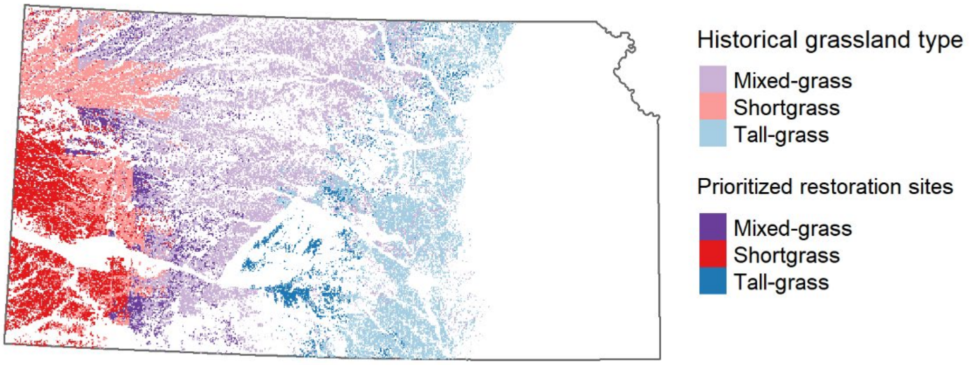

Random Forest SSP 1-2.6

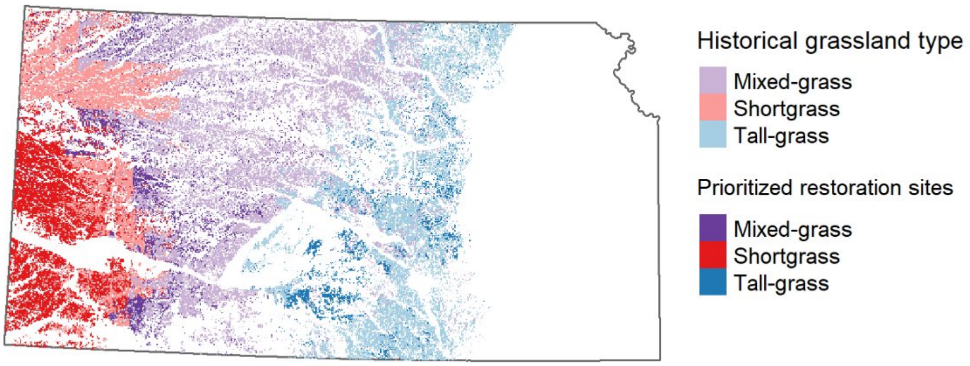

Figure S5: Priority restoration sites for the SSP 1-2.6 climate change scenarios for MaxEnt and Random Forest models. Results are from scenarios where z = 0.25, minimum habitat area to be restored per habitat type is 20%, resolution = 960x960 m, iterations = 3

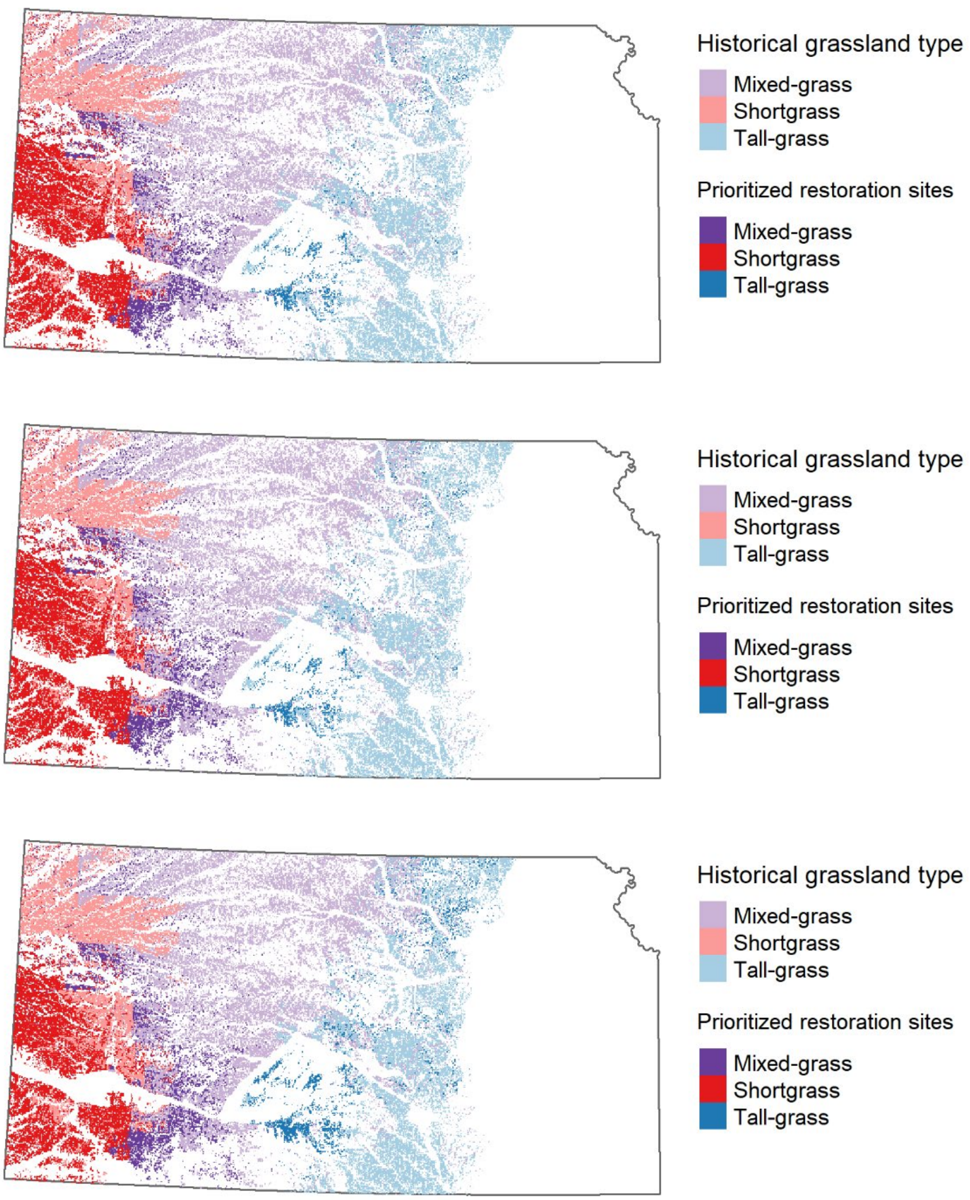

Figure S6: Priority restoration sites using the no dispersal consideration scenario when z = 0.25, resolution = 960x960 m, iterations = 3 for a minimum habitat restoration threshold of 0% (top), 10% (middle) and 20% (bottom).

A. Lesser prairie chicken (*Tympanuchus pallidicinctus*)

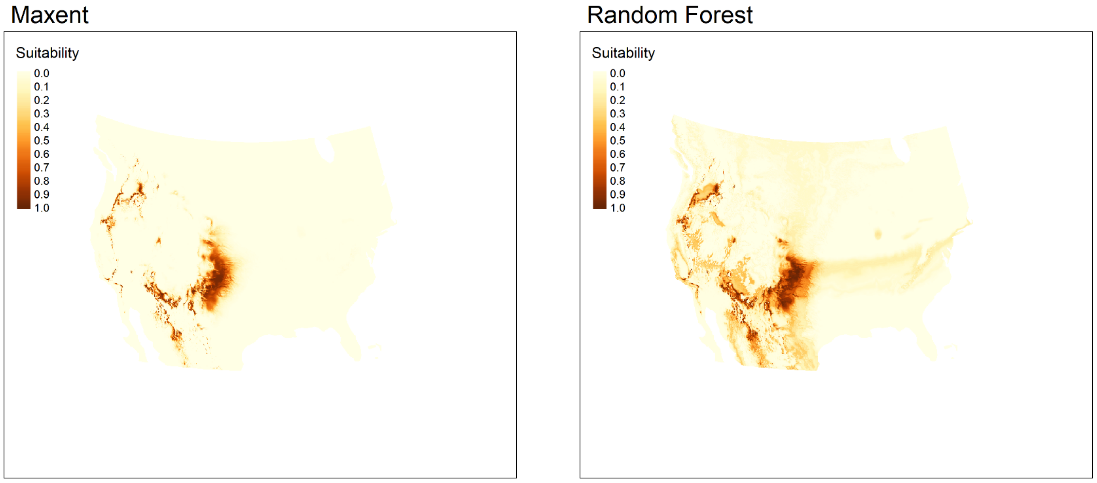

B. Greater prairie chicken (*Tympanuchus cupido*)

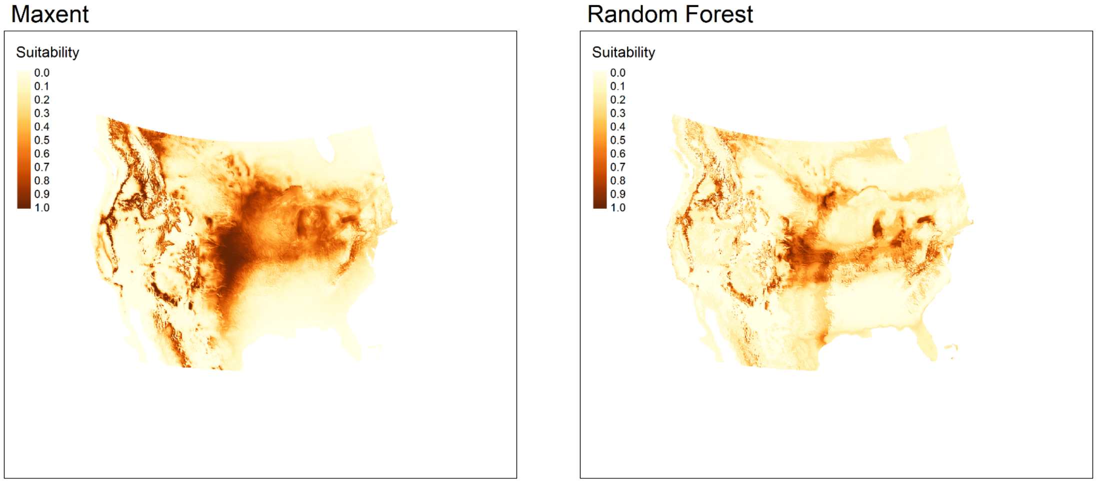

C. Swift fox (*Vulpes velox*)

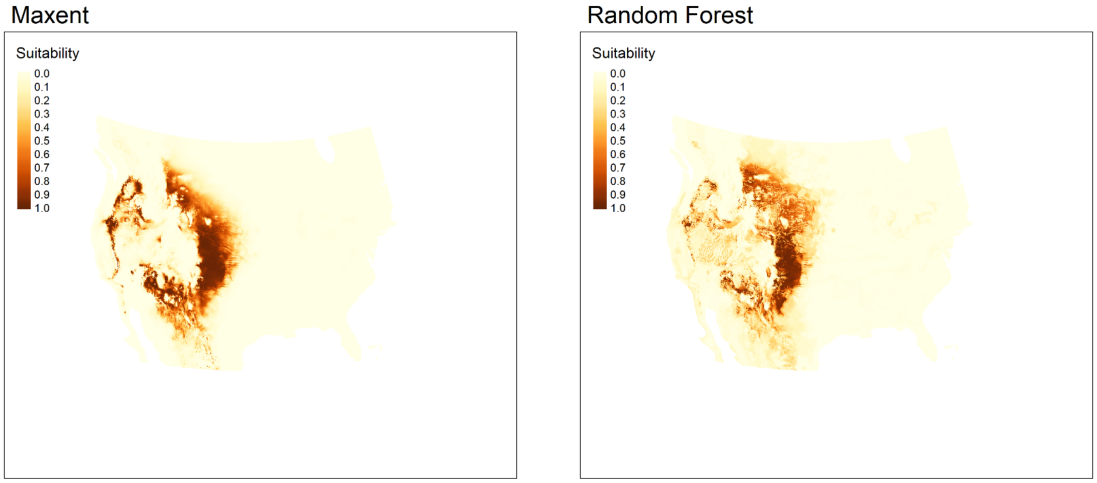

D. Pronghorn (*Antilocapra americana*)

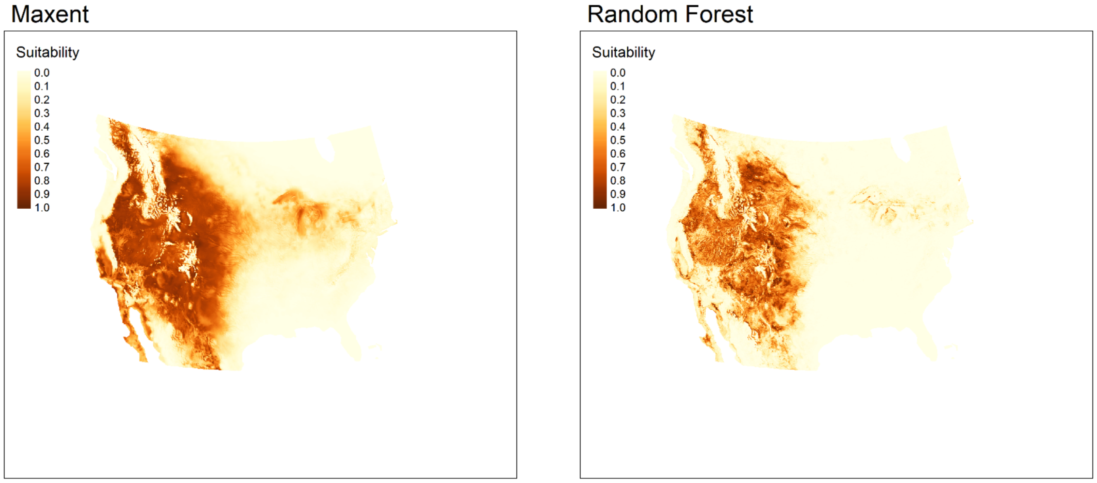

E. Regal fritillary (*Speyeria idalia*)

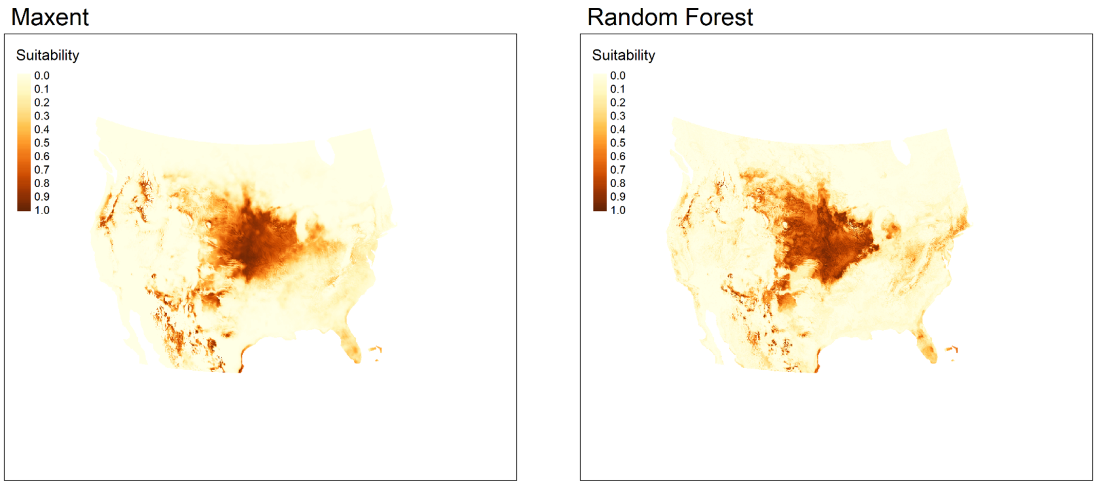

Figure S7 – Species distribution model results. For each species, we present maps of the projected suitability under SSP1-2.6 and SSP3-7.0 for MaxEnt and random forest models.

Table S1: Sources of range maps and dispersal distances for each species included in the restoration prioritization model.

**Species**	**Historical range map**	**Current range map**	**Dispersal distance**	**Habitat Type**
Swift fox (*Vulpes velox*)	(USGS and IUCN 2016)	(U.S. Geological Survey (USGS) - Gap Analysis Project (GAP) 2018a)	15 km (Moehrenschlager et al. 2004)	Shortgrass prairie, mixed-grass prairie (Moehrenschlager et al. 2004)
Pronghorn (*Antilocapra americana*)	(Kauffman et al. 2018)	(U.S. Geological Survey (USGS) - Gap Analysis Project (GAP) 2018b)	267 km (JACQUES and JENKS 2007)	Temperate grasslands (Shortgrass prairie, mixed-grass prairie, tallgrass prairie)(IUCN SSC Antelope Specialist Group 2016)
Lesser prairie chicken (*Tympanuchus pallidicinctus*)	(BirdLife International and Handbook of the Birds of the World 2008)	(U.S. Geological Survey (USGS) - Gap Analysis Project (GAP) 2018c)	50km (Hagen et al. 2004)	Shortgrass prairie, mixed-grass prairie (U.S. Fish and Wildlife Service 2021)
Greater prairie chicken (*Tympanuchus cupido*)	(BirdLife International and Handbook of the Birds of the World 2013)	(U.S. Geological Survey (USGS) - Gap Analysis Project (GAP) 2018d)	10 km (Robb and Schroeder 2005)	Tallgrass prairie (Robb and Schroeder 2005)
Regal fritillary (Speyeria idalia)	(Walker et al. 2022)	(Walker et al. 2022)	Uncertain, but some individuals able to disperse at least 3 km (Marschalek 2020, Walker et al. 2022)	Mixed-grass prairie, tallgrass prairie (Selby 2007)

Table S2: Number of observations, variables, and justifications for variable choice used in species distribution models. Bio5=mean daily maximum air temperature of the warmest month, Bio6=mean daily minimum air temperature of the coldest month, Bio13 = precipitation of the wettest month, Bio14=precipitation of the driest month.

Species	Number of observations	Variables	Justification	Average AUC from 5-fold cross validation
Lesser prairie chicken (*Tympanuchus pallidicinctus*)	30	Bio12<br>Bio6	Too few observations for more than 2 variables. Lesser prairie chickens have been found to use landscapes with <56 cm of annual precipitation (Sullins et al. 2019), so we chose Bio12. Bio6 had the highest correlation with other temperature variables.	MaxEnt = 0.99<br>Random forest = 0.99
Greater prairie chicken (*Tympanuchus cupido*)	38	Bio12<br>Bio6	Too few observations for more than 2 variables. Bio6 had the highest correlation with other temperature variables. We chose Bio12 based on preferences for the lesser prairie chicken.	MaxEnt = 0.87<br>Random forest = 0.96
Swift fox (*Vulpes velox*)	93	Bio5<br>Bio6<br>Bio13<br>Bio14<br>slope	All variables included	MaxEnt = 0.96<br>Random forest = 0.98
Pronghorn (*Antilocapra americana*)	2,675	Bio5<br>Bio6<br>Bio13<br>Bio14<br>slope	All variables included	MaxEnt = 0.872<br>Random forest =0.95
Regal fritillary (Speyeria idalia)	261	Bio5<br>Bio13<br>Bio14<br>slope	Bio5 and Bio6 were correlated. We chose Bio5 because regal fritillaries are active in the summer (Henderson et al. 2018, Post van der Burg et al. 2023).	MaxEnt = 0.921<br>Random forest = 0.95

Table S3: Area restored (km^2) for each grassland type under each restoration scenario. Results presented for multiple z-values (a metric of how biodiversity loss scales with habitat loss). See main text for descriptions of each scenario.

Grassland type	Scenario	Area restored when z=0.10	Area restored when z=0.25	Area restored when z=0.40
Mixed-grass prairie	Intermediate	5.86E+09	5.86E+09	5.86E+09
Mixed-grass prairie	Maxent126	5.86E+09	5.86E+09	5.86E+09
Mixed-grass prairie	Maxent370	6.02E+09	5.95E+09	5.87E+09
Mixed-grass prairie	Natural dispersal	5.86E+09	5.86E+09	5.86E+09
Mixed-grass prairie	No dispersal consideration	5.86E+09	5.86E+09	5.86E+09
Mixed-grass prairie	Rf126	5.86E+09	5.86E+09	5.86E+09
Mixed-grass prairie	Rf370	5.86E+09	5.86E+09	5.86E+09
Shortgrass prairie	Intermediate	1.12E+10	1.12E+10	1.12E+10
Shortgrass prairie	Maxent126	1.12E+10	1.12E+10	1.12E+10
Shortgrass prairie	Maxent370	1.1E+10	1.11E+10	1.12E+10
Shortgrass prairie	Natural dispersal	1.12E+10	1.12E+10	1.12E+10
Shortgrass prairie	No dispersal consideration	1.12E+10	1.12E+10	1.12E+10
Shortgrass prairie	Rf126	1.12E+10	1.12E+10	1.12E+10
Shortgrass prairie	Rf370	1.12E+10	1.12E+10	1.12E+10
Tall-grass prairie	Intermediate	3.33E+09	3.33E+09	3.33E+09
Tall-grass prairie	Maxent126	3.33E+09	3.33E+09	3.33E+09
Tall-grass prairie	Maxent370	3.33E+09	3.33E+09	3.33E+09
Tall-grass prairie	Natural dispersal	3.33E+09	3.33E+09	3.33E+09

Tall-grass prairie	No dispersal consideration	3.33E+09	3.33E+09	3.33E+09
Tall-grass prairie	Rf126	3.33E+09	3.33E+09	3.33E+09
Tall-grass prairie	Rf370	3.33E+09	3.33E+09	3.33E+09

Table S4: Reduced extinction risk (%) for each species under each restoration scenario. Results presented for multiple z-values (a metric of how biodiversity loss scales with habitat loss). See main text for descriptions of each scenario.

**Species**	**Scenario**	**Reduced extinction risk when z=0.10**	**Reduced extinction risk when z=0.25**	**Reduced extinction risk when z=0.40**
Greater prairie chicken	Intermediate	0.06	0.11	0.13
Greater prairie chicken	Maxent126	0.06	0.11	0.13
Greater prairie chicken	Maxent370	0.06	0.11	0.13
Greater prairie chicken	Natural dispersal	0.06	0.11	0.13
Greater prairie chicken	No dispersal consideration	0.06	0.11	0.13
Greater prairie chicken	Rf126	0.06	0.11	0.13
Greater prairie chicken	Rf370	0.06	0.11	0.13
Lesser prairie chicken	Intermediate	0.86	1.83	2.50
Lesser prairie chicken	Maxent126	0.86	1.83	2.50

Lesser prairie chicken	Maxent370	0.86	1.83	2.50
Lesser prairie chicken	Natural dispersal	0.86	1.83	2.50
Lesser prairie chicken	No dispersal consideration	0.86	1.83	2.50
Lesser prairie chicken	Rf126	0.86	1.83	2.50
Lesser prairie chicken	Rf370	0.86	1.83	2.50
Pronghorn	Intermediate	0.09	0.19	0.26
Pronghorn	Maxent126	0.09	0.19	0.26
Pronghorn	Maxent370	0.09	0.19	0.26
Pronghorn	Natural dispersal	0.09	0.19	0.26
Pronghorn	No dispersal consideration	0.09	0.19	0.26
Pronghorn	Rf126	0.09	0.19	0.26
Pronghorn	Rf370	0.09	0.19	0.26
Regal fritillary	Intermediate	0.18	0.36	0.46
Regal fritillary	Maxent126	0.18	0.36	0.46
Regal fritillary	Maxent370	0.19	0.37	0.46
Regal fritillary	Natural dispersal	0.18	0.36	0.46
Regal fritillary	No dispersal consideration	0.18	0.36	0.46
Regal fritillary	Rf126	0.18	0.36	0.46
Regal fritillary	Rf370	0.18	0.36	0.46
Swift fox	Intermediate	0.18	0.41	0.61

Swift fox	Maxent126	0.18	0.41	0.61
Swift fox	Maxent370	0.18	0.41	0.61
Swift fox	Natural dispersal	0.18	0.41	0.61
Swift fox	No dispersal consideration	0.18	0.41	0.61
Swift fox	Rf126	0.18	0.41	0.61
Swift fox	Rf370	0.18	0.41	0.61

References

BirdLife International and Handbook of the Birds of the World. 2008. Tympanuchus pallidicinctus. The IUCN Red List of Threatened Species. Version 2022-2. https://www.iucnredlist.org/.

BirdLife International and Handbook of the Birds of the World. 2013. Tympanuchus cupido. The IUCN Red List of Threatened Species. Version 2022-2. https://www.iucnredlist.org/.

Hagen, C. A., B. E. Jamison, K. M. Giesen, T. Z. Riley, A. Christian, E. Brent, M. Kenneth, Z. Terry, G. Managing, C. A. Hagen, B. E. Jamison, K. M. Giesen, and T. Z. Riley. 2004. Guidelines for Managing Lesser Prairie-Chicken Populations and Their Habitats. Wildlife Soci 32:69–82.

Henderson, R. A., J. Meunier, and N. S. Holoubek. 2018. Disentangling effects of fire, habitat, and climate on an endangered prairie-specialist butterfly. Biological Conservation 218:41–48.

IUCN SSC Antelope Specialist Group. 2016. Antilocapra americana. The IUCN Red List of Threatened Species 2016.

JACQUES, C. N., and J. A. JENKS. 2007. Dispersal of Yearling Pronghorns in Western South Dakota. Journal of Wildlife Management 71:177–182.

Kauffman, M. J., J. E. Meacham, H. Sawyer, A. Y. Steingisser, W. J. Rudd, and E. Ostlind. 2018. Wild Migrations: Atlas of Wyoming's Ungulates. Oregon State University Press.

Marschalek, D. A. 2020. Sex-biased recapture rates present challenges to quantifying population sizes and dispersal behavior of the regal fritillary butterfly (Speyeria idalia). Journal of Insect Conservation 24:891–899.

Moehrenschlager, A., B. L. Cypher, K. Ralls, R. List, and M. A. Sovada. 2004. Comparative ecology and conservation priroties of swift and kit foxes. Pages 185–198 *in* D. W.

Macdonal and C. Sillero-Zubiri, editors. Biology and conservation of wild canids. Oxford University Press, Oxford, United Kingdom.

Nolte, C. 2020. High-resolution land value maps reveal underestimation of conservation costs in the United States. Proceedings of the National Academy of Sciences of the United States of America 117:29577–29583.

Post van der Burg, M., G. MacDonald, T. Hefley, and J. Glassberg. 2023. Point-scale habitat and weather patterns influence the distribution of regal fritillaries in the central United States. Ecosphere 14:e4429.

Robb, L. A., and M. A. Schroeder. 2005. Greater Prairie-Chicken (Tympanuchus cupido): A Technical Conservation Assessment. USDA Forest Service, Rocky Mountain Region.

Selby, G. 2007. Regal Fritillary (Speyeria idalia Drury): A Technical Conservation Assessment. USDA Forest Service, Rocky Mountain Region:52.

Sullins, D. S., D. A. Haukos, J. M. Lautenbach, J. D. Lautenbach, S. G. Robinson, M. B. Rice, B. K. Sandercock, J. D. Kraft, R. T. Plumb, J. H. Reitz, J. M. S. Hutchinson, and C. A. Hagen. 2019. Strategic conservation for lesser prairie-chickens among landscapes of varying anthropogenic influence. Biological Conservation 238:108213.

U.S. Fish and Wildlife Service. 2021. Species Status Assessment Report for the Lesser Prairie-Chicken (Tympanuchus pallidicinctus), Version 2.2:110.

U.S. Geological Survey (USGS) - Gap Analysis Project (GAP). 2018a. Swift Fox (Vulpes velox) mSWFOx_CONUS_2001v1 Habitat Map. U.S. Geological Survey data release.

U.S. Geological Survey (USGS) - Gap Analysis Project (GAP). 2018b. Pronghorn (Antilocapra americana) mPRONx_CONUS_2001v1 Habitat Map. U.S. Geological Survey data release.

U.S. Geological Survey (USGS) - Gap Analysis Project (GAP). 2018c. Lesser Prairie-chicken (Tympanuchus pallidicinctus) bLEPCx_CONUS_2001v1 Habitat Map. U.S. Geological Survey data release.

U.S. Geological Survey (USGS) - Gap Analysis Project (GAP). 2018d. Greater Prairie-chicken (Tympanuchus cupido) bGRPCx_CONUS_2001v1 Habitat Map. U.S. Geological Survey data release.

USGS, and IUCN. 2016. Vulpes velox. The IUCN Red List of Threatened Species. Version 2022-2. https://www.iucnredlist.org.

Walker, A., E. Geest, and E. Royer. 2022. Argynnis idalia. https://dx.doi.org/10.2305/IUCN.UK.2022-1.RLTS.T20515A125885993.en.
