# Supplementary material for: Restoration based on cost–benefit optimization: A grasslands pilot study
Source: Ecol Appl. 2026 Jan 29;36(1):e70174. doi: 10.1002/eap.70174 (PMC12854812; doi:10.1002/eap.70174)
Supplement: Supplementary file 2 — Appendix S2. [file EAP-36-e70174-s002.pdf]

Appendix S2

Restoration based on cost–benefit optimization: A grasslands pilot study

Sarah R. Weiskopf, Toni Lyn Morelli, Tina G. Mozelewski, Alexey N. Shiklomanov, Susannah B. Lerman

Ecological Applications

ODMAP Protocol

Overview

Authorship

Contact : sweiskopf@usgs.gov

Model objective

Model objective: Forecast and transfer

Target output: Continuous habitat suitability index

Focal Taxon

Focal Taxon: Tympanuchus pallidicinctus, Tympanuchus cupido, Vulpes velox, Antilocapra americana, Speyeria idalia

Location

Location: North America and Kansas, USA

Scale of Analysis

Spatial extent: -130, -70, 25, 55 (xmin, xmax, ymin, ymax)

Spatial resolution: 30 arc sec

Temporal extent: 1981-2020 for historical species occurrence data. 1981-2010 for historical climate data. 2041-2070 for projected climate data.

Temporal resolution: 30 years

Boundary: Boundary selected to encompass historical range of selected species

Biodiversity data

Observation type: GBIF

Response data type: presence-only, point occurrence

Predictors

Predictor types: climatic, topographic

Hypotheses

Hypotheses: We hypothesize that mean annual temperature and annual precipitation are important for setting the bounds of the species' climatic niches.

Assumptions

Model assumptions: Species fill their niche and do not occur elsewhere. Species data are free from observational bias. Predictors are estimated without error. Species retain their niches across space and time. Relationship fitted under current conditions apply when transferring predictions.

Algorithms

Modelling techniques: randomForest, maxent

Model complexity: Models use different bioclimatic variables based on species. Greater and lesser prairie chickens had too few observations to include more than 2 variables. For other species, we removed correlated variables and included slope. We account for landscape factors in the next portion of our modeling exercise, and our goal is only to highlight places that are climatically suitable for the species.

Model averaging: Models were not averaged. Included both random forest and maxent outputs in the next model and compared results.

Workflow

Model workflow: 1) Download biodiversity and climate data. Filter as described above. 2) Check climate variables for correlations and remove correlated variables. 3) Convert all data to EPSG:5070 (NAD83/CONUS Albers) for an equal-area projection. 4) Fit Maxent model using default settings. 5) Fit RandomForest model. Selected 10,000 random background points and extracted predictor values for those points. Ran using classification trees (i.e., presence or absence included as a factor). Ntree=1000. Used downsampling randomForest as recommended and described by (Valavi et al. 2021) to account for imbalanced number of presence and background points. I.e., each CT used the same number of background samples as presence

samples by sampling from the background points with replacement. 6) Assess AUC with 5-fold cross-validation 7) Predict species distributions using future climate data. 8) Crop predictions to Kansas for use in restoration model.

Software

Software: R version 4.0.4 Hijmans RJ, Phillips S, Leathwick J, Elith J (2023). *dismo: Species Distribution Modeling*. R package version 1.3-14, https://CRAN.R-project.org/package=dismo. Liaw and M. Wiener (2002). Classification and Regression by randomForest. R News 2(3), 18–22. R Core Team (2023). *R: A Language and Environment for Statistical Computing*. R Foundation for Statistical Computing, Vienna, Austria. https://www.R-project.org/.

Code availability: *https://doi.org/10.5066/P14TDJUR*

Data availability: *https://doi.org/10.5066/P139JZZK*

Data

Biodiversity data

Taxon names: Tympanuchus pallidicinctus, Tympanuchus cupido, Vulpes velox, Antilocapra americana, Speyeria idalia

Taxonomic reference system: GBIF Backbone Taxonomy

Ecological level: species

Data sources: Downloaded observations from GBIF on 9/13/2024

Sampling design: Multiple - used observations from GBIF

Sample size: • Antilocapra americana 2675 • Speyeria idalia 261 • Tympanuchus cupido 38 • Tympanuchus pallidicinctus 30 • Vulpes velox 93

Clipping: North America

Scaling: Thinning distance set to 1 observation per 30 arc-sec grid cell

Cleaning: Used presence only observations from GBIF. We excluded fossil and living specimens, observations without coordinates, and observations with reported coordinate uncertainty larger than 5km or less than 3m. We used coordinate cleaner to exclude coordinates that had equal latitude and longitude, zero coordinates, or sea coordinates, and those located at country capitals, country centroids, biodiversity institutions, or GBIF headquarters.

Absence data: No absence data

Background data: Background data randomly selected from the modelled area.

Errors and biases: Because we used GBIF records for this analysis, detection probability, georeferencing errors, and sampling bias are all possible biases in the data. We tried to address these issues by excluding lower quality records and thinning occurrence records.

Data partitioning

Training data: 5-fold cross-validation. Groups set using kfold function in R.

Validation data: 5-fold cross-validation. Groups set using kfold function in R.

Test data: We did not have truly independent data, and tested our model using 5-fold cross-validation.

Predictor variables

Predictor variables: Lesser prairie chicken (Tympanuchus pallidicinctus) - Bio12, Bio6 Greater prairie chicken (Tympanuchus cupido) - Bio12, Bio6 Swift fox (Vulpes velox) - Bio5, Bio6, Bio13, Bio14, slope Pronghorn (Antilocapra americana) - Bio5, Bio6, Bio13, Bio14, slope Regal fritillary (Speyeria idalia) -Bio5, Bio13, Bio14, slope

Data sources: CHELSA V.2.x data should be cited as: Scientific publication: Karger, D.N., Conrad, O., Böhner, J., Kawohl, T., Kreft, H., Soria-Auza, R.W., Zimmermann, N.E., Linder, H.P. & Kessler, M. (2017) Climatologies at high resolution for the earth's land surface areas. Scientific Data 4, 170122. https://doi.org/10.1038/sdata.2017.122

Danielson, J.J., and Gesch, D.B., 2011, Global multi-resolution terrain elevation data 2010 (GMTED2010): U.S. Geological Survey Open-File Report 2011–1073, 26 p.

GBIF.org (13 September 2024) GBIF Occurrence Download https://doi.org/10.15468/dl.weq6mr

Spatial extent: -130, -70, 25, 55 (xmin, xmax, ymin, ymax)

Spatial resolution: 30 arc sec

Coordinate reference system: EPSG:5070

Temporal extent: 1981-2020 for historical species occurrence data. 1981-2010 for historical climate data

Temporal resolution: We used 30-year average climatology (1981-2010)

Data processing: Converted to EPSG:5070 (NAD83 / Conus Albers).

Errors and biases: Unknown

Dimension reduction: Tested for correlations among climate variables and removed variables with correlation +/- 0.8.

Transfer data

Data sources: CHELSA V.2.x data should be cited as: Scientific publication: Karger, D.N., Conrad, O., Böhner, J., Kawohl, T., Kreft, H., Soria-Auza, R.W., Zimmermann, N.E., Linder, H.P. & Kessler, M. (2017) Climatologies at high resolution for the earth's land surface areas. Scientific Data 4, 170122. https://doi.org/10.1038/sdata.2017.122

Danielson, J.J., and Gesch, D.B., 2011, Global multi-resolution terrain elevation data 2010 (GMTED2010): U.S. Geological Survey Open-File Report 2011–1073, 26 p.

Spatial extent: -102.0518, -94.58839, 36.99302, 40.00317 (xmin, xmax, ymin, ymax)

Spatial resolution: 30 arc sec

Temporal extent: 2041-2070

Temporal resolution: We used 30-year average climatology.

Models and scenarios: Climate model: GFDL-ESM4. Used CHELSA downscaled data for SSP126 and SSP370

Data processing: Converted to EPSG:5070 (NAD83 / Conus Albers).

Quantification of Novelty: We calculated multivariate environmental similarity surfaces for both climate scenarios using the 'mess' function in the dismo

Model

Variable pre-selection

Variable pre-selection: For species with limited observations, we reduced the number of variables included in the model.

Multicollinearity

Multicollinearity: Climate variables were assessed for collinearity. Removed highly correlated variables (>0.8).

Model settings

randomForest: ntree (1000), mtry (default), maxnodes (NULL), try (NA), notes (Used downsampling RF as recommended and described by (Valavi et al. 2021) to account for imbalanced number of presence and background points. Each CT used the same number of background samples as presence samples by sampling from the background points with replacement.)

<maxent>

Model settings (extrapolation): None

Model estimates

Parameter uncertainty: randomForest model was run with 1000 trees.

Model selection - model averaging - ensembles

Model averaging: Not used - considered each model separately

Model ensembles: Not used- considered each model separately

Analysis and Correction of non-independence

Spatial autocorrelation: Not assessed

Temporal autocorrelation: Not assessed

Nested data: Not assessed

Threshold selection

Threshold selection: No threshold selection used

Assessment

Performance statistics

Performance on training data: AUC

Performance on validation data: AUC

Performance on test data: NA - no truly independent data

Plausibility check

Expert judgement: Map display

Prediction

Prediction output

Prediction unit: Predictions of relative probability of presence expressed on a continuous scale.

Post-processing: Clipped output to the state of Kansas. Reprojected to EPSG:42303 to include in restoration model

Uncertainty quantification

Algorithmic uncertainty: We compared output from two different modeling algorithms.

Input data uncertainty: Not assessed

Scenario uncertainty: Ran multiple climate scenarios

Novel environments: We included novel environments in our prediction, but note that this is an assumption of the model.
